# Supplementary material for: Future climate change shaped by inter-model differences in Atlantic meridional overturning circulation response
Source: Nat Commun. 2021 Jun 16;12:3659. doi: 10.1038/s41467-021-24015-w (PMC8209213; doi:10.1038/s41467-021-24015-w)
Supplement: Supplementary file 1 — Supplementary Information [file 41467_2021_24015_MOESM1_ESM.pdf]

Supplementary Material for:

Future climate change shaped by inter-model differences in Atlantic Meridional Overturning Circulation response

Authors:

Katinka Bellomo[1*], Michela Angeloni[2,1], Susanna Corti[3], Jost von Hardenberg[4,1]

Affiliations:

[1] National Research Council of Italy, Institute of Atmospheric Sciences and Climate (CNR-ISAC), Turin, Italy

[2] Department of Physics and Astronomy, Alma Mater Studiorum - University of Bologna, Bologna, Italy

[3] National Research Council of Italy, Institute of Atmospheric Sciences and Climate (CNR-ISAC), Bologna, Italy

[4] Department of Environment, Land and Infrastructure Engineering, Politecnico di Torino, Turin, Italy

*corresponding author: k.bellomo@isac.cnr.it

Supplementary Figure 1: Dependence of AMOC change on the mean AMOC strength.
Supplementary Figure 2: Dependence of global mean air temperature change on AMOC change.
Supplementary Figure 3: Mixed-layer depth change associated with AMOC.
Supplementary Figure 4: Near-surface air temperature change associated with AMOC.
Supplementary Figure 5: Zonal mean air temperature change associated with AMOC.
Supplementary Figure 6: Estimates of mean AMOC strength.
Supplementary Table 1: List of CMIP5 and CMIP6 models.
Supplementary Table 2: Large and small AMOC decline groups.

Figures

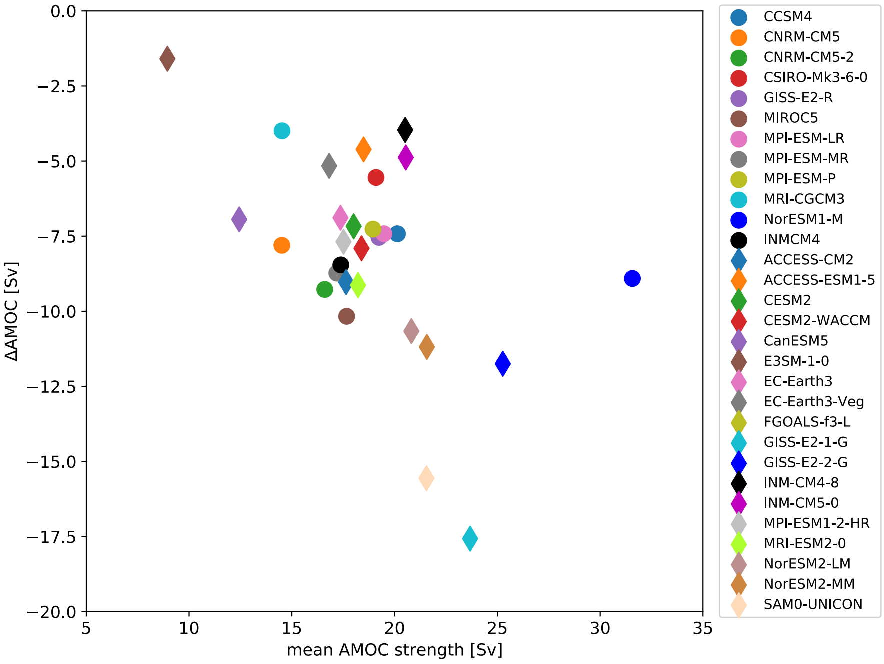

Supplementary Figure 1: Dependence of AMOC change on the mean AMOC strength. Scatterplot of the changes in AMOC (abrupt-4xCO2 minus pre-industrial control) against mean AMOC (pre-industrial control) for all models. Circles represent CMIP5 models, while diamonds represent CMIP6 models.

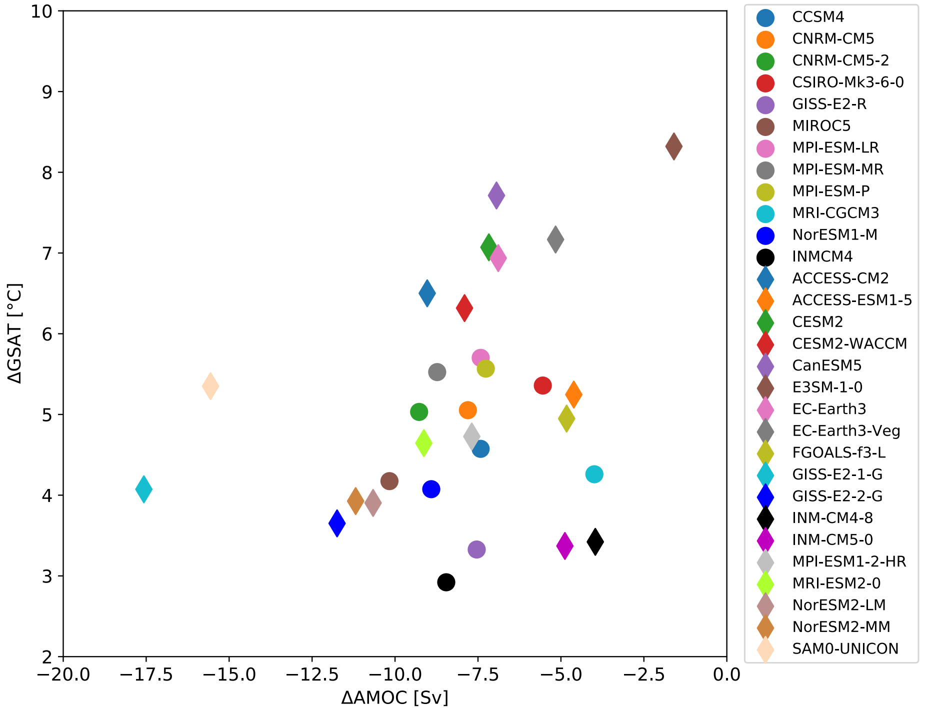

Supplementary Figure 2: Dependence of global mean air temperature change on AMOC change. Scatterplot of change in Global mean Surface Air Temperature (ΔGSAT) against change in AMOC (abrupt-4xCO_2 minus pre-industrial control) for all models. Circles represent CMIP5 models, while diamonds represent CMIP6 models.

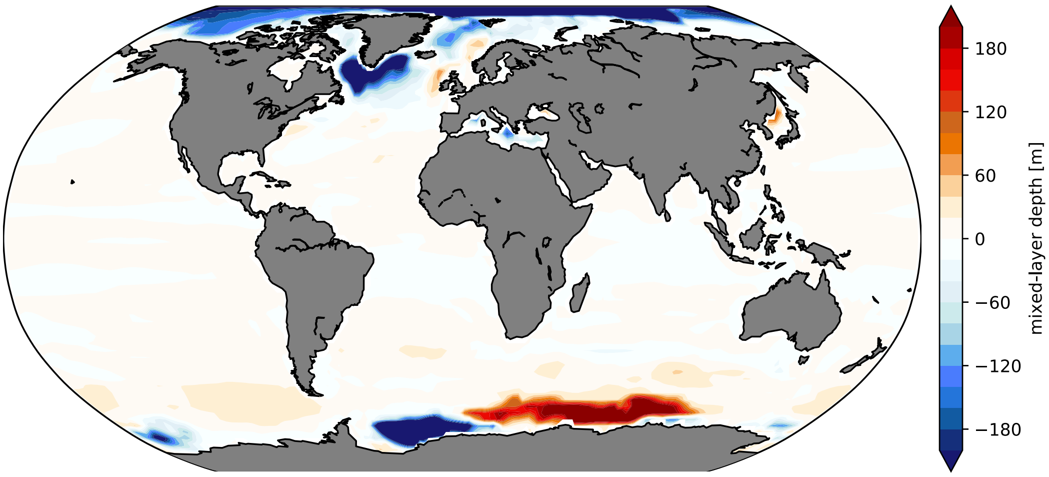

Supplementary Figure 3: Mixed-layer depth associated with AMOC. Difference in mixed-layer depth change between the averages of the large and small AMOC decline groups. Because for many models the variable of the mixed-layer depth was missing, to produce this figure we used 8 models from the large AMOC decline group (the 2 missing are: MIROC5 and SAM0-UNICON) and 7 models from the small AMOC decline group (the 3 missing are: FGOALS-f3-L, INM-CM4-8, and INM-CM5-0).

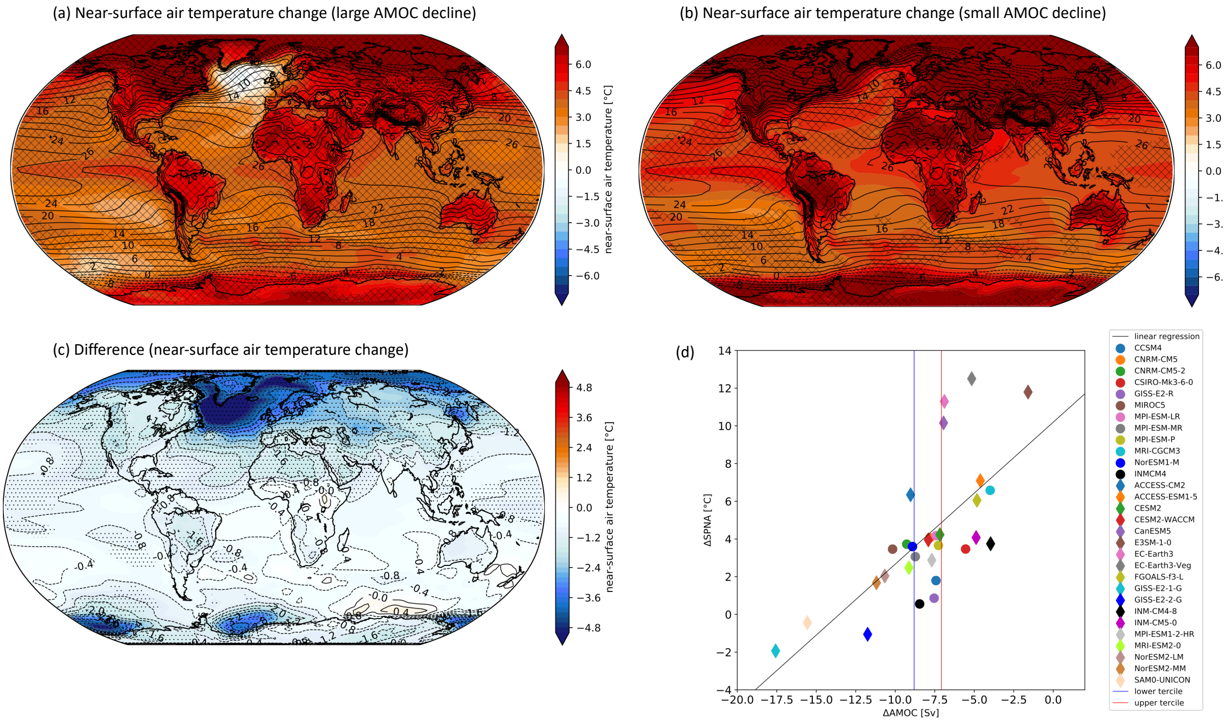

Supplementary Figure 4: Near-surface air temperature change associated with AMOC. Panels (a) and (b) show the annual mean near-surface (usually at 2 meters) air temperature change in the abrupt-4xCO2 with respect to the pre-industrial control for (a) the average of the large AMOC decline group and (b) the average of the small AMOC decline group. Panel (c) is their difference (a minus b). Units are °C. In panels (a) and (b), superimposed contours show the climatological mean near-surface air temperature computed from all models. In panel (c), superimposed contours show the numerical values associated with the near-surface air temperature differences of the colored contours. Panel (d) shows the change in sub-polar North Atlantic SST change (ΔSPNA) in units of °C against AMOC change (ΔAMOC) in units of Sv. Circles represent CMIP5 models, while diamonds represent CMIP6 models. The black line is the linear regression ($y = 0.75x + 10.19$ with R^2: 0.48), and the correlation coefficient (0.69) is statistically significant. The blue and red vertical lines represent the lower and upper terciles of the ΔAMOC distribution. Models to the left of the blue line belong to the large AMOC decline group, while models to the right of the red line belong to the small AMOC decline group.

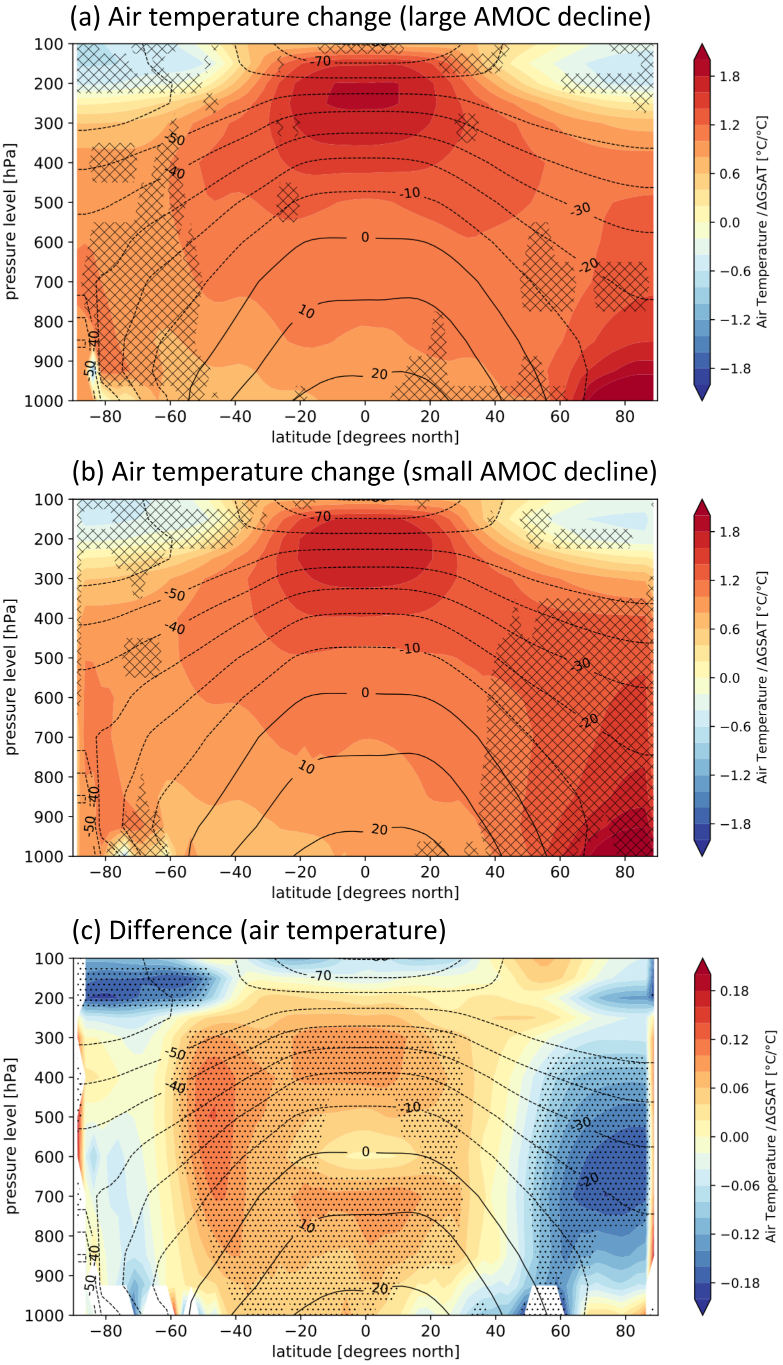

Supplementary Figure 5: Zonal mean air temperature change associated with AMOC. Panels (a) and (b) show the normalized zonal mean air temperature change of the abrupt-4xCO2 from the pre-industrial control for (a) the average of the large AMOC decline group and (b) the average of the small AMOC decline group. Panel (c) is their difference (a minus b). For each model, air temperature is divided by the respective ΔGSAT, hence the units are of °C per degree of global warming. In panels (a), (b) and (c), superimposed contours show the climatological mean zonal mean air temperature computed from all models.

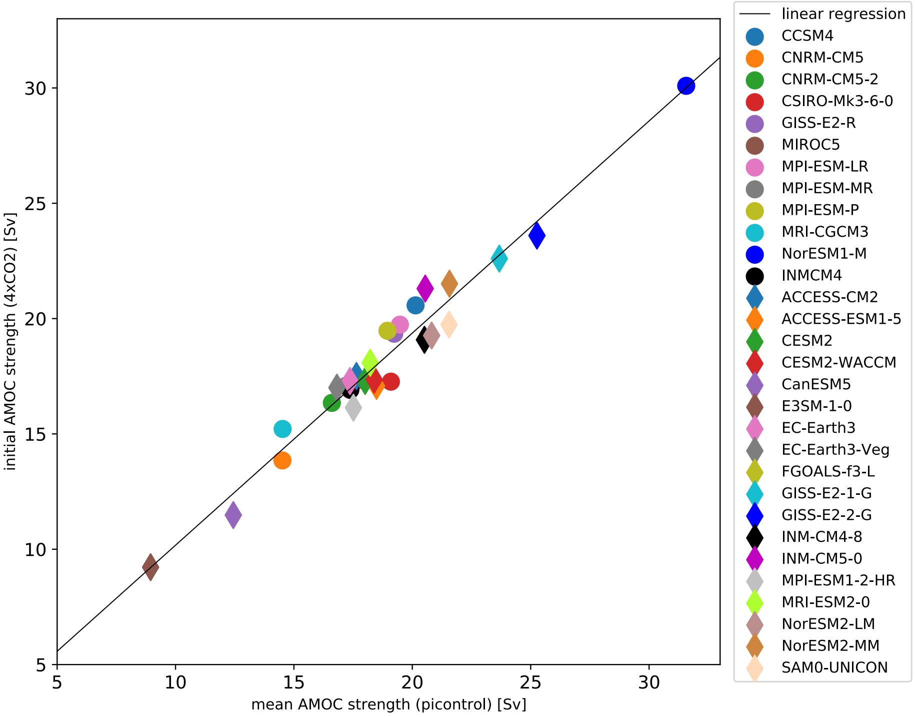

Supplementary Figure 6: Estimates of mean AMOC strength. Scatterplot of the annual mean AMOC in the first year of the abrupt-4xCO2 (initial AMOC) against mean AMOC computed from the pre-industrial control over the years 50-199 of the pre-industrial simulation for each model. Circles represent CMIP5 models, while diamonds represent CMIP6 models. The black line is the linear regression ($y = 0.92x + 0.98$ with R^2: 0.96), and the correlation coefficient (0.98) is statistically significant.

Tables

	Model	Archive	Mean AMOC strength [Sv]	Mean AMOC strength 4xCO2 [Sv]	ΔAMOC [Sv]	ΔAMOC [%]	Sub-Polar North Atlantic SST change (ΔSPNA) [°C]	Global mean Surface Air Temperature change (ΔGSAT) [°C]	ΔSPNA/ΔGSAT
1	CCSM4	CMIP5	20.14	12.72	-7.42	-37%	1.79	4.57	0.39
2	CNRM-CM5	CMIP5	14.51	6.71	-7.80	-54%	4.06	5.05	0.80
3	CNRM-CM5-2	CMIP5	16.60	7.33	-9.27	-56%	3.73	5.03	0.74
4	CSIRO-Mk3-6-0	CMIP5	19.10	13.55	-5.55	-29%	3.47	5.36	0.65
5	GISS-E2-R	CMIP5	19.24	11.70	-7.54	-39%	0.86	3.33	0.26
6	MIROC5	CMIP5	17.67	7.50	-10.17	-58%	3.46	4.17	0.83
7	MPI-ESM-LR	CMIP5	19.48	12.06	-7.42	-38%	4.17	5.70	0.73
8	MPI-ESM-MR	CMIP5	17.19	8.46	-8.73	-51%	3.06	5.52	0.55
9	MPI-ESM-P	CMIP5	18.95	11.69	-7.26	-38%	3.66	5.57	0.66
10	MRI-CGCM3	CMIP5	14.52	10.53	-3.99	-27%	6.58	4.26	1.55
11	NorESM1-M	CMIP5	31.57	22.66	-8.91	-28%	3.60	4.07	0.88
12	inmcm4	CMIP5	17.38	8.93	-8.46	-49%	0.56	2.92	0.19
13	ACCESS-CM2	CMIP6	17.64	8.61	-9.03	-51%	6.34	6.50	0.98
14	ACCESS-ESM1-5	CMIP6	18.49	13.88	-4.61	-25%	7.09	5.25	1.35
15	CESM2	CMIP6	18.01	10.84	-7.17	-40%	4.23	7.07	0.60
16	CESM2-WACCM	CMIP6	18.39	10.49	-7.90	-43%	3.95	6.32	0.63
17	CanESM5	CMIP6	12.44	5.50	-6.94	-56%	10.16	7.71	1.32
18	E3SM-1-0	CMIP6	8.95	7.35	-1.59	-18%	11.79	8.32	1.42
19	EC-Earth3	CMIP6	17.37	10.48	-6.89	-40%	11.29	6.94	1.63
20	EC-Earth3-Veg	CMIP6	16.82	11.66	-5.16	-31%	12.50	7.17	1.74
21	FGOALS-f3-L	CMIP6	19.48	14.65	-4.83	-25%	6.05	4.95	1.22
22	GISS-E2-1-G	CMIP6	23.67	6.10	-17.57	-74%	-1.93	4.07	-0.47
23	GISS-E2-2-G	CMIP6	25.26	13.52	-11.75	-46%	-1.05	3.65	-0.29
24	INM-CM4-8	CMIP6	20.51	16.55	-3.96	-19%	3.75	3.42	1.09
25	INM-CM5-0	CMIP6	20.55	15.67	-4.88	-24%	4.08	3.37	1.21
26	MPI-ESM1-2-HR	CMIP6	17.51	9.83	-7.69	-44%	2.88	4.73	0.61
27	MRI-ESM2-0	CMIP6	18.22	9.09	-9.13	-50%	2.47	4.64	0.53
28	NorESM2-LM	CMIP6	20.82	10.16	-10.66	-51%	2.04	3.90	0.52
29	NorESM2-MM	CMIP6	21.57	10.38	-11.19	-52%	1.68	3.93	0.43
30	SAM0-UNICON	CMIP6	21.55	5.99	-15.56	-72%	-0.44	5.35	-0.08
	Min		8.95 Sv	5.50 Sv	-1.59 Sv	-18%	-1.93 °C	2.92 °C	-0.47
	Max		31.57 Sv	22.66 Sv	-17.57 Sv	-74%	12.50 °C	8.32 °C	1.74

Supplementary Table 1: List of CMIP5 and CMIP6 models.

	Model	Archive	Mean AMOC strength [Sv]	AMOC strength 4xCO2 [Sv]	ΔAMOC [Sv]	ΔAMOC [%]	Subpolar North Atlantic SST change (ΔSPNA) [°C]	Global mean Surface Air Temperature change (ΔGSAT) [°C]	ΔSPNA/ΔGSAT
1	CSIRO-Mk3-6-0	CMIP5	19.10	13.55	-5.55	-29%	3.47	5.36	0.65
2	MRI-CGCM3	CMIP5	14.52	10.53	-3.99	-27%	6.58	4.26	1.55
3	ACCESS-ESM1-5	CMIP6	18.49	13.88	-4.61	-25%	7.09	5.25	1.35
4	CanESM5	CMIP6	12.44	5.50	-6.94	-56%	10.16	7.71	1.32
5	E3SM-1-0	CMIP6	8.95	7.35	-1.59	-18%	11.79	8.32	1.42
6	EC-Earth3	CMIP6	17.37	10.48	-6.89	-40%	11.29	6.94	1.63
7	EC-Earth3-Veg	CMIP6	16.82	11.66	-5.16	-31%	12.50	7.17	1.74
8	FGOALS-f3-L	CMIP6	19.48	14.65	-4.83	-25%	6.05	4.95	1.22
9	INM-CM4-8	CMIP6	20.51	16.55	-3.96	-19%	3.75	3.42	1.09
10	INM-CM5-0	CMIP6	20.55	15.67	-4.88	-24%	4.08	3.37	1.21
11	CNRM-CM5-2	CMIP5	16.60	7.33	-9.27	-56%	3.73	5.03	0.74
12	MIROC5	CMIP5	17.67	7.50	-10.17	-58%	3.46	4.17	0.83
13	NorESM1-M	CMIP5	31.57	22.66	-8.91	-28%	3.60	4.07	0.88
14	ACCESS-CM2	CMIP6	17.64	8.61	-9.03	-51%	6.34	6.50	0.98
15	GISS-E2-1-G	CMIP6	23.67	6.10	-17.57	-74%	-1.93	4.07	-0.47
16	GISS-E2-2-G	CMIP6	25.26	13.52	-11.75	-46%	-1.05	3.65	-0.29
17	MRI-ESM2-0	CMIP6	18.22	9.09	-9.13	-50%	2.47	4.64	0.53
18	NorESM2-LM	CMIP6	20.82	10.16	-10.66	-51%	2.04	3.90	0.52
19	NorESM2-MM	CMIP6	21.57	10.38	-11.19	-52%	1.68	3.93	0.43
20	SAM0-UNICON	CMIP6	21.55	5.99	-15.56	-72%	-0.44	5.35	-0.08

Supplementary Table 2: Large and Small AMOC decline groups. Top 10 (rows 1 through 10): list of the models belonging to the small AMOC decline group; Bottom 10 (rows 11 through 20): list of the models belonging to the large AMOC decline group.
